# Improvements in docking scoring functions: the physics-based perspective

**DOI:** 10.1186/1758-2946-4-S1-O22

**Published:** 2012-05-01

**Authors:** Xavier Barril

**Affiliations:** 1 Dept. Fisicoquímica, Facultat de Farmàcia, Av. Joan XXIII, s/n 08028 - Barcelona, Spain

## 

Docking is possibly the most widely used technique in structure-based drug design. It is recognized as extremely useful to guide the development of active molecules as well as in virtual screening to identify new leads. But docking is also notoriously imperfect and many aspects need to improve. In this talk I will present our work on two different sources of errors in the docking predictions: protein-ligand interaction potentials and internal energy of the ligands. The use of higher-level calculations offers an opportunity to obtain better quality results and offers a glimpse of the full potential of the technique, but introducing this information back into fast and general scoring functions remains a challenge.

